# Chronic non-communicable diseases and the challenge of universal health coverage: insights from community-based cardiovascular disease research in urban poor communities in Accra, Ghana

**DOI:** 10.1186/1471-2458-14-S2-S3

**Published:** 2014-06-20

**Authors:** Ama de-Graft Aikins, Mawuli Kushitor, Kwadwo Koram, Stella Gyamfi, Gbenga Ogedegbe

**Affiliations:** 1 Regional Institute for Population Studies, University of Ghana, P.O.Box LG96, Legon, Ghana; 2 Noguchi Memorial Institute for Medical Research, University of Ghana, P.O.Box LG581, Legon, Ghana; 3 Ussher Polyclinic, Ghana Health Service, P.O.Box GP2105, Accra, Ghana; 4 Division of Health and Behavior, Center for Healthful Behavior Change, New York University School of Medicine, New York, NY, USA

## Abstract

**Background:**

The rising burden of chronic non-communicable diseases in low and middle income countries has major implications on the ability of these countries to achieve universal health coverage. In this paper we discuss the impact of cardiovascular diseases (CVD) on primary healthcare services in urban poor communities in Accra, Ghana.

**Methods:**

We review the evidence on the evolution of universal health coverage in Ghana and the central role of the community-based health planning services (CHPS) programme and the National Health Insurance Scheme in primary health care. We present preliminary findings from a study on community CVD knowledge, experiences, responses and access to services.

**Results:**

The rising burden of NCDs in Ghana will affect the achievement of universal health coverage, particularly in urban areas. There is a significant unmet need for CVD care in the study communities. The provision of primary healthcare services for CVD is not accessible, equitable or responsive to the needs of target communities.

**Conclusions:**

We consider these findings in the context of the primary healthcare system and discuss the challenges and opportunities for strengthening health systems in low and middle-income countries.

## Background

The rising burden of chronic non-communicable diseases in low and middle income countries (LMICs), like Ghana, has major implications for the ability of these countries to achieve universal health coverage. We define universal health coverage (UHC) in this paper as the provision of primary healthcare services that are accessible, equitable and responsive to the needs of target communities [1]. Two key challenges have been identified. First, many of these countries have a double burden of infectious and chronic diseases. This dual burden weakens health systems that are already compromised. Current knowledge suggests that strong health systems must have the following six building blocks, which must work synergistically to provide universal health coverage: service delivery, human resources, medicines and technology, information systems, financing and governance. For many LMICs, health systems lack some or most of the elements of the building blocks. Sub-Saharan Africa (SSA), for example has 11% of the world’s population, bears over 24% of global disease burden, but only has 3% of the global health workforce [2]. The growing burden of NCDs poses further challenges to a limited and poorly distributed health workforce. Second, the impact of NCDs on communities is great. NCDs affect adults in their most productive age, affect poor communities disproportionately, push individuals and households further into poverty and present long-term psychosocial challenges [3,4]. Emerging evidence suggests that the burden of NCDs is growing among the urban poor in low and middle income countries [5]. Poor communities experience a dual burden of infectious and chronic diseases and poor health and life outcomes due to limited access to healthcare. There has been a new focus on what these challenges mean for achieving UHC, with attention focused on financing healthcare and strengthening the health workforce, as well as addressing broader issues of equity and rights [6-8].

## Methods

This paper draws on insights from a community-based CVD project which, as one of its objectives, aims to examine the feasibility of task-shifting CVD care to community health workers to improve the quality and reach of CVD care within the primary healthcare system in these communities. Task shifting is defined as the rational distribution of tasks among health workforce teams [9,10]. It is especially useful in low-income settings facing healthcare human resource crisis, such as SSA countries, which have the most acute healthcare workforce shortage in the world. In order to maximize the efficient use of health workforce resources, healthcare tasks are shifted from higher-trained health workers to less skilled health workers. When feasible, task shifting can occur among physicians, non-physician clinicians, nurses, and community health workers. Successful CVD task-shifting interventions have been reported in Cameroon and South Africa [9].

The paper is presented in three parts. In the first part we provide a brief history of UHC in Ghana and focus on two major primary healthcare interventions that have sought to improve primary healthcare for marginalised communities: the community-based health planning and services (CHPS) programme and the National Health Insurance Scheme (NHIS). In the second part we provide a synthesis of the evidence of Ghana’s NCD burden and its impact on UHC particularly in urban poor communities. In the third part we describe our longitudinal work in three urban poor communities in Accra which has focused on measuring prevalence of hypertension and diabetes and gathering mixed (quantitative and qualitative) data on CVD knowledge, experiences and responses at household, community and health systems levels. To conclude we consider the opportunities and challenges inherent in developing community-based interventions for CVD that can strengthen primary healthcare services for CVD and other prevalent conditions in urban areas.

## Results

### Universal health coverage in Ghana

Ghana’s Ministry of Health developed a 10-year plan for establishing and implementing primary healthcare services (PHCs) in 1977. A year later the government signed up to the 1978 Alma-Ata agreement. PHCs have been a key focus in healthcare delivery in the country since then. Within this framework are two central strategies. The first strategy has been the provision of community-based health planning and services (CHPS). The CHPS programme adopted in 1999, started as a pilot community-based health intervention programme in Northern Ghana [11]. The CHPS programme has been adopted by 104 of Ghana’s 110 districts. It focuses on remote and underserved areas of the country and involves using trained community health workers to provide curative and preventive care, typically immunizations, family planning and minor health issues. Initially CHPS services were provided in designated community health centres called CHPS compounds. Now there is an emphasis on mobile service provision involving door-to-door prevention and treatment services. This new dimension of CHPS has been adapted in urban poor communities where space rarely exists for the construction of CHPS compounds and where communities are highly mobile.

The second PHC strategy has been the National Health Insurance Scheme (NHIS). Developed and implemented in 2003, the NHIS aimed to provide financial protection for Ghanaians as part of a broad objective to achieve universal health coverage. By 2010, only 34% of Ghanaians had subscribed to the scheme. There have been challenges in increasing subscriber numbers, targeting the poor for exemptions, and ensuring that healthcare provider costs are controlled [12-14]. Currently there are NHIS pilots underway that seek to improve cost containment and control cost escalation by sharing risk between schemes, healthcare providers and subscribers.

PHC delivery in Ghana has identified challenges. Experts note that a lack of finances, inequitable distribution of services and weak collaboration between health and development sectors undermine the quality and reach of PHC [15,16]. There is persistent over-utilization of secondary and tertiary healthcare services because of weak gate-keeping at the level of community healthcare. Quality of care at PHC level is poor and community perceptions of care are unfavourable. Finally primary healthcare facilities are evenly distributed across the rich-poor divide and are not particularly pro-poor. These challenges are further compounded by Ghana’s rising burden of NCDs and the complex long-terms demands these conditions pose for community-level interventions.

### Ghana’s burden of NCDs and impact on urban poor communities

Chronic non-communicable diseases (NCDs) have become major causes of disability and death in Ghana. Hypertension and CVDs represent a significant proportion of this NCD burden. Urban hypertension prevalence is 32.3%, while rural prevalence is 27% [17]. In contrast HIV prevalence is 1.8%. Prevalence rates of major risk factors for NCDs – especially poor diets, overweight/obesity physical inactivity, alcohol over-consumption - are high. Hypertension is the fifth commonest cause of outpatient morbidity nationally. In the Greater Accra Region, where our community-based project is situated, hypertension is the second cause of outpatient mortality. The Ghanaian NCD burden reflects the SSA situation. While infectious diseases still account for at least 69% of deaths, age specific mortality rates from chronic diseases as a whole are higher in SSA than virtually all other regions of the world [18]. It is estimated that 75% of deaths in SSA will be attributable to hypertension by the year 2020 [19].

There is significant unmet need for the prevention, treatment and care of NCDs. Ghana’s healthcare system struggles to cope with a double burden of infectious and chronic diseases. As a result health professionals are poorly trained in NCD diagnosis and management and lack appropriate knowledge and skills [20,21]. Health facilities lack the appropriate equipment for diagnosis, monitoring and treatment. Medicines are either expensive or unavailable. Competitive traditional medicine and faith healing systems offer unregulated chronic disease care to both urban and rural communities [21]. Lay and patient knowledge of NCDs is poor. Community-based prevalence surveys consistently show that up to 70% of individuals living with hypertension or diabetes do not know they have these conditions [22]. This leads to late presentations at medical facilities, healer-shopping (between biomedicine, ethnomedicine and faith healing) and poor self-care. This interplay of factors is implicated in high morbidity and mortality rates. At the Korle-Bu Teaching Hospital in Accra, the proportionate mortality for hypertension and its associated complications such as stroke is around 15% and most of the CVD deaths occur in the productive age group between 40 and 60 years (autopsy series, 1990 – 2000; [23]).

Poverty plays a significant role in NCD risk, morbidity and mortality in Ghana [24]. Ghana is experiencing a ‘protracted polarised’ health transition with two key elements. First, populations have lived with a protracted co-existence of infectious and chronic diseases over the last few decades. Second, the double burden of disease is polarized across socio-economic status. While wealthy communities experience higher risk of chronic diseases, poor communities experience higher risk of infectious diseases and a ‘double jeopardy’ of infectious and chronic diseases. Poor communities are also more likely to develop complications and die prematurely from their conditions due to poor access to medical care and their daily association with health disabling environments.

Research shows that urban poverty in SSA presents a powerful barrier to health-promoting behaviours, regardless of country [25]. Poor communities experience health disadvantage at multiple stages: from “the person’s beliefs about health and disease, and actual behaviour, to presentation, screening, risk assessment, negotiation, participation, programme persistence and treatment adherence” [26]. The multiple health disadvantages of the urban poor will place a great toll on an already overburdened health infrastructure and undermine the goals of UHC.

There is a gap between policy recognition of Ghana’s NCD problem and the development and implementation of appropriate policies. This gap needs to be addressed. Policymakers recognized that Ghana had a chronic disease problem in the early 1990s [22]. Hypertension and diabetes were included on a ‘priority health intervention list’, and a Non-Communicable Disease Control Programme (NCDCP) was established. Important developments have occurred in the last 10 years: (1) the Ministry of Health (MOH) has introduced a Regenerative Health and Nutrition Programme, which aims to address five dimensions of preventive health: diet, water intake, exercise, rest, and sanitation [27]; (2) the NHIS includes some medicines for hypertension, diabetes and cancers on its payment exemption list. (3) a draft NCD policy has been developed and it places emphasis on prevention as a key dimension of reducing healthcare costs at the levels of individuals, families, health systems and government; (4) the MOH participated in a UN High Level Meeting on NCDs in September 2011 and signed up to a ‘whole of society, whole of government’ approach to tackling the NCD crisis in LMICs. What is urgently needed is the development of practical intervention models that can be aligned to existing models of healthcare, such as the current PHC strategies of community-based care and financial protection for the urban and rural poor.

### Primary healthcare in urban poor communities

There are three main challenges to the provision of primary healthcare to urban poor communities. First, high population density and mobility characterising urban poor communities present challenges for access and uptake of primary healthcare services. The traditional CHPS approach, for example, has been difficult to implement for this reason. The new mobile approach to CHPS aims to address the challenge of public access by taking healthcare to the doorstep of community members. The second challenge is the limited public health focus of PHC. CHPS involves using trained community health workers to provide curative and preventive care, typically on immunizations, family planning and minor health issues. The intervention does not focus on NCDs, which constitute a growing public health threat to urban poor communities. Finally, although the NHIS aims to provide financial protection for poor and vulnerable Ghanaians, methods for targeting the poor have been problematic and have led to some deserving poor – such as the urban poor – being excluded from exemptions. Thus, in communities such as our study communities, individuals living with long-term chronic conditions pay out of pocket for medical care.

### A community-based research project

The Regional Institute for Population Studies (RIPS), with support from the Secretariat of the African Caribbean and Pacific Group of States – ACP-EU Cooperation Programme in Higher Education (EDULINK) and IDRC, has established an active research field site in three urban poor communities in Accra: Jamestown, Ussher Town and Agbobloshie. This long-term project includes longitudinal Urban Poverty and Health Surveys to examine health, poverty and development indicators in these settings over time and to provide vital data to local and regional stakeholders for the development of the communities. The project has also incorporated a longitudinal mixed method study on CVD experiences and care in the community, which is supported by a collaborative New York University-University of Ghana (NYU-UG) grant. The aim of the CVD study is to gather baseline and long-term data on community knowledge, experiences and responses to CVD, as well as health systems responses to CVD in the communities and to develop community-based CVD interventions.

Jamestown, Ussher Town and Agbobloshie are formally labelled poor by the Accra Metropolitan Assembly because of their low socio-economic status compared to the national average [28]. The communities are characterized by high population density, low socio-economic status and a built-up environment with poor sanitary conditions and poor housing structures. The average monthly household income in all three communities is only GHC126.13 (USD 78.83) [28]. This places the communities in the 4^th^ and lowest income class within the Accra Metropolitan Area: the average monthly income of the 1^st^ income class ranges from GHC500 to GhC1250 ($312.5 - $781.25) and the 3^rd^ income class ranges from GHC99.12 and GHC137.38 ($61.95 - $85.86). About three-quarters of the population have attained up to Junior High School (or middle school) education and above; however the quality of education is reported to be low with a dominance of poorly resourced public schools and there is more male school enrolment compared to female. James Town and Ussher Town are inhabited mainly by the Ga-Dangme ethnic group. The main economic activities in these communities are fishing and petty trading. Agbogbloshie is an ethnically heterogeneous and migrant community with most inhabitants working as traders and artisans.

### Health status of the study community

Three survey rounds for the main Urban Poverty and Health Survey have been conducted at 18-20 month intervals in 2010 (June), 2011 (December) and 2013 (September), with households and individuals. The qualitative study on cardiovascular disease (diabetes and hypertension) knowledge and experiences was nested within the survey rounds. Round 1 focused on 497 households and 736 individuals aged between 15 and 98. Round 2 focused on 806 households and 947 individuals aged between 15 and 98. Round 2 also included measurements of blood pressure (BP) and body-mass index (BMI) of 617 individuals using standard epidemiological methods. Round 3 involved BP and BMI measurements, as well as fasting blood glucose and cholesterol measurements. Preliminary analysis of the Round 2 survey and measurements of BP and BMI showed that the research communities were high risk populations for cardiovascular disease. The overall prevalence of hypertension in all three communities was 28.3% with higher prevalence among men (31.0%) compared to women (25.6%). Over a third of the study population were either overweight or obese with higher rates in women than in men. Crucially, levels of awareness and treatment of hypertension were poor. Among respondents who had hypertension, only 7.4% were aware of their condition and 62.9% perceived they had no risk. Furthermore only 4% were on antihypertensive medication and only 3.5% of hypertensive individuals had adequate blood pressure (BP) control [29]. Three key insights emerged from data gathered on lifestyle practices and illness management. First, the relationship between high salt, high fat foods, alcohol and hypertension and diabetes was not commonly known. Secondly, lifestyle practices were poor: physical inactivity was common; consumption of fruits and vegetables was low, consumption of processed fats was high and snacking on processed foods was common. These lifestyle practices were linked to the lack of adequate spaces for regular physical activity and an obesogenic environment with a dominant presence of fast foods and processed foods on most community streets. Finally, there were gendered differences in exposure to risk and illness management. Knowledge of hypertension and diabetes symptoms was poor particularly among women. The level of awareness and treatment of hypertension was lower in men than in women (3.1% and 1.3% for men and 11.9% and 6.5% for women, respectively) and the rate of control was higher among women compared with men (5.0% and 2.1%, respectively) [29]. These preliminary findings showed important associations between socio-economic context, gender and CVD risk in the study communities. The findings also showed a significant unmet need for CVD care in the communities.

### Primary healthcare delivery to the study community

**Figure 1:**
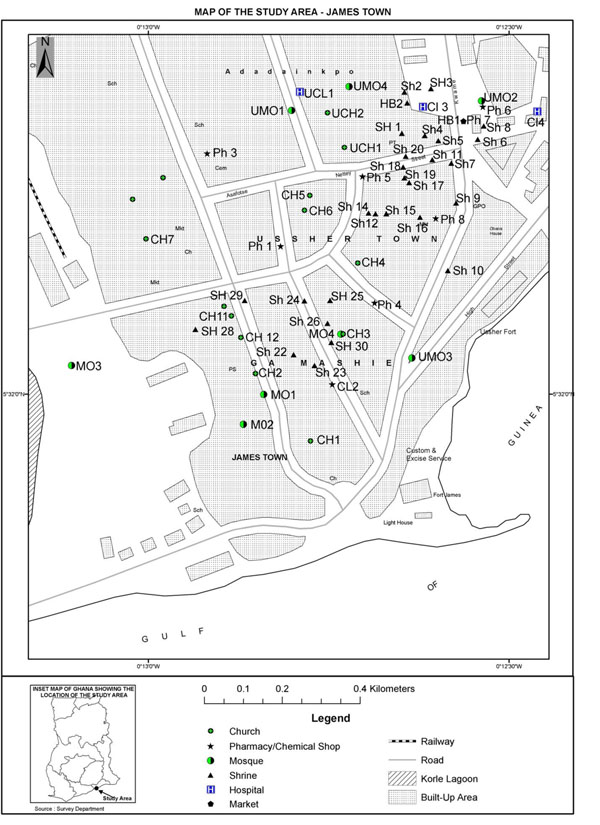
Map of the study area

The study communities are served by pluralistic medical systems including a government polyclinic, private health centres, pharmacies and chemical shops, traditional herbal shops and religious spaces (churches, mosques) that offer faith-based healing. Figure 1 presents the spatial profile of health services in two research communities: James Town and Ussher Town. Table 1 presents a breakdown of the numbers of services per type of service. Private and traditional healthcare services outnumber public sector health services in the research communities. Furthermore faith-based spaces – churches, mosques and shrines – far outnumber health centres. Churches and mosques provide legitimate healthcare for individuals and are particularly crucial for providing psychosocial support during prolonged periods of illness and other crises [30,31].

**Table 1:** Health facilities in James Town

Health Centres	Numbers
Churches	14

Clinics (private)	2

Mosques	8

Poly Clinic (public)	1

Pharmacies (private)	6

Chemical shops	3

Traditional Shrines	27

Interviews with people with diabetes and hypertension suggest that all the services, with the exception of traditional shrines, are perceived as legitimate providers of care for diabetes and hypertension and are regularly used [32]. Traditional shrines, most belonging to multi-generational families, are reserved for diagnosis and treatment of social conditions (e.g., family problems, job security, marital success). Formal biomedical services, and especially doctors, are respected for their technical competence and expert knowledge on prevalent conditions, but are viewed as inaccessible and expensive [32]. While primary healthcare through the urban CHPS programme is supposed to provide care at the doorstep of individuals, this does not happen in practice. Thus individuals tend to access care at the private pharmacies first, who then refer to the community polyclinic or the teaching hospital located a short distance away from the communities. Individuals shop around for healers throughout the process of acute and chronic treatment, accessing alternative health solutions from other medical spaces (outside the community), herbal remedies and faith healing. Our preliminary analysis of diabetes experiences showed that few individuals (seven out of 20 respondents) had subscribed to the National Health Insurance Scheme. Poverty was the dominant reason provided for opting out of NHIS. For the minority who had NHIS subscription, challenges still existed with access to prescribed medications that were not on the NHIS exemption list, laboratory tests and diabetes care products such as blood-glucose meters [32]. There was also an emerging mistrust of the quality of NHIS endorsed medicines prescribed by doctors, with a number of individuals referring to NHIS medicines as counterfeit medicines.

We examined the feasibility of task-shifting CVD care among community health workers (hereafter CHWs) in the public and private health sectors. We recruited 32 CHWs from the polyclinic, private clinics, pharmacies, and chemical and herbal shops. They belonged to three categories of health workers: (1) community health nurses (CHNs) who were engaged mainly in family planning, maternal and child health care; (2) health promotion officers whose main aim was to assist the CHNs and to provide general education on health and sanitation to community members; and (3) community health workers who worked for pharmacies, chemical and herbal shops and engaged in diagnosis, treatment and referral to primary, secondary and tertiary health services. Of these three categories of health workers only the herbalists stated expertise in CVD care: one stated their ability to help people with diabetes and hypertension, regain their sexual vitality. Table 2 presents the profile of respondents.

**Table 2:** Socio-demographic details of health workers

	Total number of respondents
*Sex*	
Female	16
Male	16

*Age*	
<30	20
31-40	3
41-50	3
51-60	1
>60	1
Non-response	4

*Education*	
No education	2
Primary	-
Middle	1
Secondary	17
Tertiary	12

*Categories of service*	
Private sector – clinics	2
Private sector – pharmacies	3
Public sector - polyclinic	23
Chemical shops	1
Herbal shops/traditional medicine	3

*Duration of Service*	
<1year	16
1 – 5 years	6
6-10years	1
>10 years	6
Non-responses	3

We conducted individual interviews which explored their perceptions and knowledge of health, illness, chronic illness, diabetes, hypertension and stroke. We also explored their perspectives on health systems challenges relating to NCD care in the community and sought their recommendations for improving these challenges. We tested their objective knowledge of CVD risks, symptoms and management using a CVD knowledge questionnaire adapted to test the knowledge of lay health workers in faith-based organisations in Accra [30]. We have also used the questionnaire to test CVD knowledge within the communities at household level and in faith-based organizations. Here we focus solely on the results of the CVD knowledge survey.

Community health workers lacked fundamental knowledge on CVD risk, prevention, control and treatment. Areas on which the majority of health workers demonstrated poor knowledge included: the relationship between high red meat consumption and poor health, diabetes as a risk factors for heart disease, the relationship between family history and individual risk of heart disease, and the need for individuals with hypertension to use blood pressure medicine for life. There were important differences in knowledge across the different categories of health workers. Community health nurses had the highest total knowledge score. This group had been healthcare providers for a long period. The health promotion officers had the lowest knowledge score. This group had been newly hired during the time of the interviews and collectively had limited knowledge of NCDs generally and of CVD in particular. A key aspect of task-shifting is to improve the knowledge of conditions, including risk factors, diagnostic aspects, treatment and treatment outcomes. Thus a major dimension of our task-shifting project will be to educate the CHWs on CVD risk, diagnosis, treatment and treatment outcomes as a first step towards building capacity for improved CVD care at the community level.

## Discussion

Ghana, like many LMICs, has complex public health challenges. The rising prevalence of NCDs creates further challenges for the country’s health system and its focus on achieving UHC. We have discussed preliminary evidence on the impact of CVD on PHC services in urban poor communities. We have shown that NCDs are a major problem in urban poor communities in Ghana, that there is a significant unmet need for CVD care in these communities. The provision of PHC services for CVD is not accessible, equitable or responsive to the needs of target communities. Public health services are outnumbered by private healthcare facilities, as well as by faith-based organizations that provide faith-based healing. There is a strong tendency for community members to bypass the public health facilities and to access care in private pharmacies and clinics or to access care from public facilities but shop around for healers across the wide range of health services. This deepens the existing problem in Ghana of public overutilization of secondary and tertiary health services [33]. There is poor capacity for CVD care in the public PHC facilities. While the urban CHPS programme is in operation, CHWs focus largely on maternal and child health problems, which constitute only part of a complex public health burden. CHW knowledge on crucial aspects of CVD risk, symptoms and treatment is poor and this has major implications for their ability to educate communities and to assist in the diagnosis, treatment and continuity of care for those living with CVD. This is also likely to deepen the existing problem of poor public perceptions of the technical competence of PHC workers [33]. Poor public perceptions have been implicated in the over-utilization of secondary and tertiary health services, as well as complementary and alternative medicine.

Despite these challenges, opportunities exist for creating synergies between PHC and NCD prevention strategies. CHPS and NHIS provide important frameworks to incorporate NCD care. CHWs have been trained to provide care in dominant community health problems. Similar training on CVD can enhance knowledge, confidence and ability to provide rudimentary care and support for people with CVD. This approach has worked in the Cameroonian and South African contexts [9]. Ghana’s NCD burden was not originally considered in the development of the NHIS in 2003. Ten years on NCDs have been placed on the NHIS agenda, with exemptions for a limited number of CVD medicines. What is needed in the future is attention to the other products of NCD care such as laboratory tests and NCD care products (e.g. blood glucose meter, BP monitors). The new efforts to target deserving beneficiaries of free healthcare, such as the ultra-poor, will need to incorporate the specific needs of poor urban individuals living with CVD and other chronic conditions [13,14].

The focus on empowering communities to minimize risk will be crucial to lessening the burden of NCDs on PHC services. People lie at the heart of health systems, as recipients and producers of healthcare [1]. NCDs present important financial and psychosocial challenges for affected individuals, families, households and communities. Research in other LMICs suggest that the significant burden NCDs pose for health systems necessitates a move towards full self-management of chronic life-long conditions through strategies such as expert patient networks [34]. Patient empowerment (through expert patient networks) presupposes the existence of strong and evolved health systems that are committed to the ideology of patient-centred care and conscientized communities of patients that exercise their power to make beneficial healthcare choices. These elements then enable reciprocal non-hierarchical interactions between patients and healthcare providers aimed at enhancing the long-term health and quality of life of patients. This scenario is rare in SSA due to health systems deficiencies. In Accra, for example, there is a growing number of patient networks that aim to provide basic education and psychosocial support to members [21]. However, due to a lack of resources and political visibility, their impact on psychosocial outcomes has not been as strong as that of expert patient networks in high income countries. The challenge lies in empowering patient networks and relevant community groups to enhance the role of communities in successful primary healthcare delivery.

## Conclusions

The Ghanaian case study presented here reflects a growing challenge posed by NCDs to PHC delivery in other LMICs. Studies from Cameroon, Liberia, India and Malaysia, for example, highlight the rising burden of NCDs in urban poor contexts, the inability of health systems to address the complex care implications of NCD experiences and the need for health systems strengthening that prioritizes the role of people with NCDs, and their families and communities [5,6,34-37]. These studies suggest the need for a systems approach to NCD management. Such an approach requires that healthcare delivery systems are redesigned to respond to the long term nature of NCDs. Models developed specifically for the under-resourced and fragmented health systems of LMICs such as the Innovative Care for Chronic Conditions Framework focus on the need to improve healthcare delivery by developing multi-level synergies between patient and families at the micro-level, community and healthcare organization at the meso level and coordinated policy and health systems at the macro level. There is a consensus that the participatory role of people in primary and secondary prevention of NCDs is critical to the development of effective long-term interventions in PHC.

## Competing Interests

All authors had no competing interests to declare.

## Authors’ contributions

The article was conceptualised by all authors. AdGA drafted the article. MK contributed to the data synthesis. All authors read and approved of the final version.

## Declarations

The study on task-shifting CVD care to community health workers was supported by a collaborative New York University-University of Ghana (NYU-UG) grant.

This article has been published as part of *BMC Public Health* Volume 14 Supplement 2, 2014: Responsive health systems: working with the community on control of non-communicable diseases (NCDs). The full contents of the supplement are available online at http://www.biomedcentral.com/bmcpublichealth/supplements/14/S2.
